# Formulation and In Vitro Evaluation of Matrix Tablets Containing Ketoprofen–Beta Cyclodextrin Complex for Enhanced Rheumatoid Arthritis Therapy: Experimental and Computational Insights

**DOI:** 10.3390/pharmaceutics17040474

**Published:** 2025-04-05

**Authors:** Monica Stamate Cretan, Lacramioara Ochiuz, Vlad Ghizdovat, Monica Molcalut, Maricel Agop, Carmen Anatolia Gafițanu, Alexandra Barsan (Bujor), Mousa Sha’at, Ciprian Stamate

**Affiliations:** 1 Department of Pharmaceutical Technology, Faculty of Pharmacy, “Grigore T. Popa” University of Medicine and Pharmacy, 16 Universitatii Street, 700115 Iasi, Romania; monica.stamate@umfiasi.ro (M.S.C.); carmengafitanu@gmail.com (C.A.G.); alexandra.m.bujor@umfiasi.ro (A.B.); mousa-shaat@umfiasi.ro (M.S.); 2 Biophysics and Medical Physics Department, “Grigore T. Popa” University of Medicine and Pharmacy, 16 Universitatii Street, 700115 Iasi, Romania; vlad.ghizdovat@umfiasi.ro; 3 Faculty of Physics, “Alexandru Ioan Cuza” University, Blvd. Carol I, No. 11, Iasi 700506, Romania; monica.molcalut@uaic.ro; 4 Department of Physics, “Gheorghe Asachi” Technical University of Iasi, Prof. Dr. Docent Dimitrie Mangeron Rd., No. 59A, 700050 Iasi, Romania; magop@tuiasi.ro; 5 Romanian Scientists Academy, 54 Splaiul Independentei, 050094 Bucharest, Romania; 6 Department of Mechanical Engineering, Mechatronics and Robotics, “Gheorghe Asachi” Technical University of Iasi, Prof. Dr. Docent Dimitrie Mangeron Rd., No. 43, 700050 Iasi, Romania; cip101301@gmail.com

**Keywords:** compressed tablets, ketoprofen, beta cyclodextrin, drug complexes, mathematical models, dissolution test

## Abstract

**Background:** Rheumatoid arthritis is a chronic autoimmune disease that leads to severe disability and requires improved therapeutic strategies to optimize anti-inflammatory treatment. This study aimed to address this challenge by developing and characterizing an extended-release polymer matrix tablet containing ketoprofen and a ketoprofen–β-cyclodextrin complex with enhanced therapeutic properties. The objective was to improve inflammation management and therapeutic outcomes using a novel delivery system based on the inclusion of the active substance in cyclodextrin complexes. **Methods:** Tablets were formulated using ketoprofen and ketoprofen–β-cyclodextrin complexes combined with hydrophilic polymers such as Carbopol^®^ 971P NF, Kollidon^®^ VA 64, and Methocel^TM^ K4M. The complexes were obtained via the coprecipitation method to improve bioavailability. The kinetics of the release of ketoprofen, ketoprofen–β-cyclodextrin complex (2:1), and ketoprofen–β-cyclodextrin complex (1:1) from the tablets were investigated in vitro in artificial gastric and intestinal fluids, and drug release profiles were established. Advanced mathematical models were used to describe the nonlinear behavior of the drug–polymer systems. **Results:** The inclusion of ketoprofen in the β-cyclodextrin complexes was confirmed, revealing distinct release profiles. Tablets (K-3 F-3) containing the 1:1 complex showed rapid release (96.2% in 4–7 h), while tablets (K-1 F-4) containing free ketoprofen released 76% over 9–11 h. Higher polymer concentrations slowed the release due to gel barrier formation. Pharmacotechnical and stability tests supported their suitability as extended-release forms. A multifractal modeling approach described the release dynamics, treating the polymer–drug matrix as a complex system, with release curves characterized by variations in the fractal dimension and resolution. **Conclusions:** Specific hydrophilic polymer combinations effectively prolonged ketoprofen release. The developed matrix tablets, which were evaluated via in vitro studies and mathematical modeling, show promise for improving therapeutic outcomes and patient compliance during rheumatoid arthritis treatment.

## 1. Introduction

Joint pain, known medically as arthralgia, can affect one or more joints in the human body. Approximately ten percent of the global population suffers from joint pain, which affects all age groups, including children, adults, and the elderly. Typically, joint pain is caused by rheumatism, arthritis, or arthrosis. Rheumatoid arthritis is a chronic inflammatory disease that affects the joints, skin, eyes, lungs, heart, and blood vessels. This autoimmune disorder occurs when the body’s immune system attacks its own joint cartilage, causing painful inflammation, followed by cartilage erosion and joint deformity [1,2]. The main goals of treatment for rheumatoid arthritis are to reduce joint inflammation and pain. Treatment involves the administration of anti-inflammatory, analgesic, and corticosteroid drugs. Nonsteroidal anti-inflammatory drugs (NSAIDs) are compounds belonging to the group of anti-inflammatory, analgesic, and antipyretic agents in which the anti-inflammatory effect predominates [3,4,5,6,7,8,9]. They eliminate or reduce some of the signs and symptoms of inflammation associated with rheumatic diseases.

NSAIDs are generally weak organic acids that are relatively well absorbed and are primarily eliminated via the kidneys after hepatic metabolism. These drugs cause gastric irritation, the severity of which decreases with food intake [2,3,8,9,10,11,12,13,14].

The main objective of administering a pharmaceutical formulation in the treatment of inflammatory diseases is to deliver a sufficient amount of the active substance to the site of inflammation to achieve therapeutic effectiveness. Another goal is to reach therapeutic plasma concentrations in a timely manner and to maintain them over a defined period. These objectives can be achieved by synthesizing ketoprofen–β-cyclodextrin complexes, which improve drug release in the body, followed by their incorporation into matrix tablet formulations. The formulation of matrix tablets containing drug–cyclodextrin complexes represents an innovative approach to the treatment of rheumatoid arthritis. These tablets offer several advantages: they enable controlled release of the active substance over an extended period, reduce the dosing frequency, and improve patient compliance.

Ketoprofen (K), an active substance from the NSAID class, belongs to the group of analgesic, antipyretic, and anti-inflammatory agents in which the anti-inflammatory effect predominates [3,12,13,14]. Ketoprofen, or (RS)-2-(3-benzoylphenyl) propionic acid (C_16_H_14_O_3_, Mr = 254.281 g/mol), contains a single asymmetric carbon atom and is marketed as a racemic mixture of two enantiomers in equal proportions (50:50): the R (−) and S (+) enantiomers. From a pharmacological perspective, the S (+) enantiomer is responsible for inhibiting cyclooxygenase enzymes and is considered the main contributor to the drug’s analgesic and anti-inflammatory effects [3,4,12,13,14]. According to the literature [9,15], the solubility of ketoprofen in aqueous media varies with pH, reaching its maximum at a pH of 2.5 and its minimum—approaching zero—at a pH of 7.

**Figure 1:**
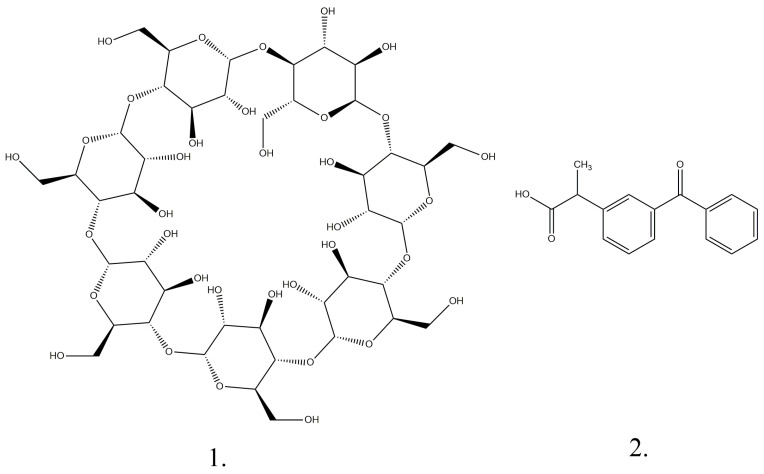
Chemical structures of β-cyclodextrin (**1**) and ketoprofen (**2**).

Ketoprofen (Figure 1) is primarily metabolized in the liver, like many other drugs. For conventional-release tablets, ketoprofen has a half-life of approximately 2 h and 15 min, while for extended-release formulations, the half-life extends to 5 h and 15 min. Therapeutic blood concentrations are typically reached 30 min after oral administration, with peak levels observed after around 2 h [4,12,13,14]. Ketoprofen also penetrates the synovial fluid, where concentrations can persist longer than in serum. In more than 80% of cases, this drug is excreted renally in the form of glucuronide conjugates. Ketoprofen therapy is particularly indicated for diseases such as acute inflammatory rheumatism, rheumatoid arthritis, ankylosing spondylitis, and osteoarthritis [2,4,12,13,14].

Ketoprofen is insoluble in water, and to increase its solubility it has been complexed with various cyclodextrins, as shown by certain studies [5,6,7]. Ketoprofen is complexed mainly with beta cyclodextrin, as shown by the data in the literature [7,8,9], and for this reason we chose beta cyclodextrin as the complexing agent in order to be able to compare our results with those in the literature.

β-Cyclodextrin (Figure 1) is a cyclic oligosaccharide composed of seven D-glucose units (Figure 1) arranged around a central hydrophilic cavity and an outer hydrophilic one with amphipathic properties ((C6H10O5)7, Mr = 1134.98 g/mol). Cyclodextrins are crystalline substances with a homogeneous structure. They are non-hygroscopic and have a toroidal shape similar to a macro ring [3,4,5,6,7,8,9,10,11,12,13,14]. The hydrophilic exterior of the cyclodextrin molecule makes this substance soluble, and the hydrophobic interior of the molecular cavity provides a favorable environment for the inclusion of nonpolar molecules [6].

Kollidon^®^ VA 64 is a vinylpyrrolidone–vinyl acetate copolymer and is frequently used as a binder for the manufacture of tablets by direct compression or wet granulation or for coating films [7,8,12,13,14]. This excipient allows tablets with excellent hardness, even under low-compression-force conditions, to be produced by direct compression, an inexpensive technological process, with minimal energy consumption and in a short time. The new assortment, which has been significantly improved, is highly miniaturized, has improved plasticity, and has an increased capacity to bind to the active substance in the dry state. Kollidon^®^VA64 is an effective binder due to its smaller dimensions and improved plasticity, allowing the binding of six molecules of an active substance or a mixed active substance with a single molecule of Kollidon. This property leads to a finished product with increased hardness and low friability, increasing mechanical stability [9,10,12,13,14]. In the formulation of tablets, it can be associated with excipients such as different types of starch, sorbitol, and microcrystalline cellulose because their binder properties are insufficient to produce tablets with very good mechanical properties [10,12,13,14].

Another polymer used to delay drug release is Carbopol^®^ 971P NF (carbomer resin). This polymer, when associated with others, forms a porous structure inside tablets. Drugs can be dispersed in the pores of this structure. In the dissolution medium, the structure hydrates, the pores enlarge, and the drug dissolves and is released. Due to these characteristics, this polymer has begun to be frequently used in prolonged-release tablets [10,11,12,13,14]. Another quality of Carbopol^®^ 971P NF is that it provides increased tablet hardness and controlled drug release [12,13,14]. A Carbopol^®^ 971P NF content of approximately 30–40% therefore leads to a zero-order release of the drug substance, considering that it is associated in the formulation with other retarding agents [11,12,13,14,16].

Hydroxypropyl methylcellulose (Methocel^TM^ K4M, C_56_H_108_O_30_, Mr = 1261.4 g/mol) is a semi-synthetic macromolecule. It is a cellulose derivative that has good wetting properties. After hydration it forms a three-dimensional network that gradually erodes and thus releases the drug [11,13,14,17].

Sodium carboxymethylcellulose (Croscarmellose) is a crosslinked polymer (C_8_H_16_NaO_8_, Mr = 263.2 g/mol) belonging to the category of hydrophilic materials. It is a semi-synthetic polymer based on cellulose that, like hydroxypropyl methylcellulose, has good wetting and dissolving properties, making it a good disintegrant, especially in the case of extended-release tablets, where the addition of retarding agents that are too strong can make it impossible for tablets to dissolve in the digestive tract. Croscarmellose helps to progressively erode tablets and release drugs from them [10,11,12,13,14,18].

Sorbitol is used in tablets to improve their textures and to hydrate their matrices. It is an excipient that is available in powder form, and it ensures free flow due to its crystalline structure. Crystalline sorbitol (Sorbidex P 16616) is a unique excipient for direct compression because it has excellent flow properties, ensuring uniform mixing and a constant weight per tablet [12,13,14,15,19]. Crystalline grades of sorbitol powder (C_6_H_14_O_6_, Mr = 182.2 g/mol) have been specifically developed. Sorbitol added to a volume of powder to be compressed improves the flow of the powder and the degree of compaction, leading to tablets with superior stability, and its sweet taste masks the unpleasant taste of the other components [10,11,12,13,14,15,19,20].

Magnesium stearate is also known as octadecanoic acid (Mg (C_18_H_35_O_2_)_2_, Mr = 591.27 g/mol). Magnesium stearate appears as a white powder and is used in tablets as a lubricating agent, interposing itself between the particles of the powder mixture and the surfaces of the compression punches and the mold. The powder will not stick to them, resulting in intact tablets without pinches [12,13,14,20,21].

The aim of this study was to develop compressed tablets using ketoprofen and ketoprofen complexes with beta cyclodextrin as the active substances. These molecular complexes were formulated as polymer matrix tablets with prolonged release, representing innovative formulations used in the treatment of rheumatoid arthritis. Six matrix tablets (F1–F6) were prepared through direct compression using ketoprofen (K-1), a ketoprofen–beta cyclodextrin (2:1) complex (K-2), a ketoprofen–beta cyclodextrin (1:1) complex (K-3), Kollidon^®^ VA 64, Carbopol^®^ 971P NF, Methocel^TM^ K4M, Croscarmellose, sorbitol, and magnesium stearate in different concentrations. For the first three tablets, formulas K-1 F-1, K-2 F2, and K-3 F-3, the excipient concentrations were 11. 2% for Kollidon^®^ VA 64, 4% for Carbopol^®^ 971P NF, 11.6% for Methocel^TM^ K4M, 11.6% for Croscarmellose, 0.8% for sorbitol, and 0.8% for magnesium stearate. For the last three tablets, formulas K-1 F-4, K-2 F5, and K-3 F-6, the excipient concentrations were 11. 2% for Kollidon^®^ VA 64, 8% for Carbopol^®^ 971P NF, 11.6% for Methocel^TM^ K4M, 11.6% for Croscarmellose, 0.4% for sorbitol, and 0.8% for magnesium stearate. The quantities chosen for the polymers fell within the ranges specified by the *Romanian Pharmacopoeia* [22] and did not exceed the maximum permitted values. Another important objective of this study was the development of mathematical models with fractals that could predict how ketoprofen was released from tablets when it was associated with different polymers, starting from in vitro studies.

## 2. Materials and Methods

### 2.1. Chemicals and Reagents

Ketoprofen, or 2-(3-benzoylphenyl)propionic acid (K, ≥98% purity), and β-cyclodextrin (β-CD, ≥97% purity) were obtained from Sigma Aldrich Chemie GmbH (Steinheim, Germany). Potassium dihydrogen phosphate (KH_2_PO_4_, ≥99.5%; Mr = 136.01 g/mol), potassium chloride (KCl, ≥99%; Mr = 74.5 g/mol), and concentrated hydrochloric acid (HCl, ≥37%; Mr = 36.46 g/mol) were obtained from Chimreactiv SRL (Bucharest, Romania). Sodium hydroxide (NaOH, 98.5%; Mr = 40.00 g/mol) and ethanol (CH_3_ CH_2_ OH, ≥96.0%; Mr = 46.07 g/mol) were purchased from Chemical Company S.A. (Iasi, Romania). Distilled water was obtained using a GFL type 2004 distillation unit (Burgweld, Germany). All reagents met the quality requirements, according to national and European standards, and were used without any purification operations. Carbopol^®^ 971P NF (carbomer resin) was obtained from The Lubrizol Corporation (Wickliffe, OH, USA). Kollidon^®^ VA 64 (copolyvidone) was obtained from Sigma Aldrich Chemie GmbH (Steinheim, Germany). Magnesium stearate (C_36_H_70_MgO_4_, magnesium content: 4.0–5.0%, Mr = 591.24 g/mol) was obtained from Union Derivan S.A (Barcelona, Spain). Methocel^TM^ K4M (C_56_H_108_O_30_, Mr = 1261.4 g/mol) was an excipient obtained from Funahai (Zhejiang, China), and Croscarmellose (C8H16NaO8, Mr = 263.2 g/mol) was obtained from Sigma Aldrich Chemie GmbH (Steinheim, Germany). Matrix tablets (K-β-CD) were based on the sustained-release polymers Carbopol^®^ 971P NF, Kollidon^®^ VA 64, Methocel^TM^ K4M, and Croscarmellose.

### 2.2. Equipment

Microscopic analysis of ketoprofen–beta cyclodextrin complexes was performed on an SEM Quanta 200 3D electron microscope (Osceola, FL, USA). For high-resolution infrared spectral analysis, the equipment used was a Bruker Optics IFS 66 series FT-IR spectrometer (Billerica, MA, USA). The DSC analysis equipment used was a Du Pont DSC auto sampler 910 Differential Scanning Calorimeter (990701) (Hixson, TN, USA). A multiposition ceramic hotplate stirrer with 3 positions was used (MHK-4-3) (65 cm width, 13 cm length, stirring speed up to 1600 rpm, and heating up to 50 °C) (Harlow, UK). pH was determined using an inoLAB pH 7110 pH meter (Xylem Analytics GmbH, Weilheim, Germany). A PIONEER^®^ analytical balance (Ohaus Corporation, Parsippany, NJ, USA), an SR 8 Plus Dissolution Test Station 73-100-104 dissolution apparatus (Hanson Re-search, Chatsworth, CA, USA), a 0.5–5.0 mL Rotilabo^®^—Mikroliterpipette (Carl Roth GmbH, Karlsruhe, Germany), and a Korsch EK0 tablet press with 9 mm flat punches and 10 kN of tableting pressure (Korsch AG, Berlin, Germany) were used.

### 2.3. Preparation of Complexes with Ketoprofen and Beta Cyclodextrin

The synthesis of inclusion compounds with cyclodextrins to enhance the solubility of poorly soluble pharmaceutical substances is based on a “host-guest” interaction in which the host cyclodextrin accepts the drug molecule as a guest and stores it in its cavity. Ketoprofen–cyclodextrin inclusion complexes can be achieved due to the molecular structure of cyclodextrin, which has a toroidal shape with a hollow interior in which medicinal substances can be included. The molecular masses of the medicinal substances must be smaller than the cavity in order to be able to penetrate this cavity [6,7,8,9,12,13,14,23]. The molecular weight of ketoprofen allows two molecules to be incorporated into the same cyclodextrin cavity. The preparation method used was coprecipitation by magnetic stirring.

In order to obtain the complexes by the method of coprecipitation by magnetic stirring, ketoprofen and beta cyclodextrin were used in the two molar ratios of 2:1 and 1:1. Cyclodextrin solutions were prepared by dissolving the required amount of β-cyclodextrin in distilled water at 50 °C for 1 h, and ketoprofen solutions were prepared by dissolving the required amount of the active substance in concentrated alcohol. The alcohol solution was added to the aqueous solution with stirring, and the resulting mixture was magnetically stirred at room temperature for 3 h until a suspension was obtained. The resulting suspension was cooled to 4 °C over 24 h. The complex formed was isolated by filtration, washed with ethanol to remove traces of uncomplexed ketoprofen, dried in an oven at 50 °C, and stored in a brown container for subsequent characterization.

### 2.4. Characterization of Complexes with Ketoprofen and Beta Cyclodextrin

The obtained ketoprofen–cyclodextrin complexes were analyzed by electron microscopy, Fourier transform infrared analysis, and thermogravimetry. For each type of analysis, the samples were processed according to the specific protocols of the devices.

For microscopic analysis, an SEM Quanta 200 3D microscope was used, and the powders were attached by gluing on metal supports. Then, they were sprayed with gold and analyzed in a vacuum chamber.

FTIR analysis was performed using a Bruker-Optics-IFS 66 FT-IR spectrometer for which the powder samples were mixed by trituration in a mortar with potassium bromide. Then, they were transformed into pellets. Scanning of the samples was performed under ambient-temperature conditions in the standard range of 4000–400 cm^−1^.

Thermogravimetric analysis was performed with a DuPont DSC-910 using argon. Heating was performed at approximately 10 °C per minute, and the flow rate was approximately 35 cc/min. Indium was used to calibrate the temperature of the device. The melting enthalpy was calculated using the area of the endothermic peak recorded on the thermogram. Samples weighing 5 g were tested over a temperature range from 20 °C to 450 °C.

Dissolution studies for both ketoprofen and its complexes with beta cyclodextrin were performed in phosphate buffer solutions with a pH of 6.8. The amount of medium used at 37 °C was 500 mL, and the stirring speed was 100 revolutions per minute. Samples of ketoprofen, ketoprofen–cyclodextrin complex (1:1), and ketoprofen–cyclodextrin complex (2:1) weighing 100 mg were subjected to analysis. Samples with volumes of approximately 3 mL were collected every 5 min, and the test lasted 60 min. The collected samples were diluted and analyzed spectrophotometrically at the ketoprofen-specific wavelength of 233 nm.

### 2.5. Development of Polymeric Matrix Tablets with Ketoprofen and Ketoprofen–Beta Cyclodextrin

In this study, six tablet formulations were developed, each containing ketoprofen as the active pharmaceutical ingredient. It was incorporated in three different forms to assess the influence of its molecular state on formulation performance. Free ketoprofen was used in formulations labeled K-1 F-1 and K-1 F-4. In formulations K-2 F-2 and K-2 F-5, ketoprofen was complexed with cyclodextrin in a 2:1 molar ratio, while in formulations K-3 F-3 and K-3 F-6, the drug was complexed with cyclodextrin in a 1:1 molar ratio. These variations were designed to evaluate the impact of complexation and the molar ratio on the physicochemical properties and potential bioavailability of the final products. The amount of ketoprofen per tablet was 70 mg, and the amounts of the ketoprofen–cyclodextrin complexes were 150 mg. For the first three tablet formulations (K-1 F-1, K-2 F-2, and K-3 F-3), Carbopol was used at a concentration of 4%, and for the last three tablet formulations (K-1 F-4, K-2 F-5, and K-3 F-6) the concentration was 8%. For all tablets, the concentrations of the following excipients were identical: 11.2% Kollidon, 0.8% magnesium stearate, 11.6% Methocel, and 11.6% Croscarmellose. Sorbitol, being used as a diluent, was added to each formula to bring the mass up to 250 mg. Tablets were obtained by compressing the powder mixtures using a Korsch EK0 tablet press equipped with cylindrical punches with diameters of 9 mm.

### 2.6. Dissolution Studies

All six tablet formulations were subjected to dissolution testing using a Pharmacopoeia-specified Apparatus II (paddle method) [22], with cylindrical vessels placed in a thermostated bath to ensure a dissolution medium with a constant temperature of 37 ± 0.5 °C. The stirring rate was set to 60 rpm, and paddle stirrers were used. The in vitro dissolution media were artificial gastric juice and artificial intestinal juice. The tablets (K-1 F-1, K-2 F-2, K-3 F-3, K-1 F-4, K-2 F-5, and K-3 F-6) were placed in the cylindrical vessels in the test solutions in turn. Thus, they spent the first two hours in the artificial gastric juice (pH = 1.2) and then up to 12 h in the artificial intestinal juice (pH = 6.8). To study the release in the artificial gastric juice, samples with volumes of 3 mL each were extracted at set time intervals as follows: half an hour, one hour, and two hours. For each amount of sample extracted, the medium was supplemented with the same amount of fresh medium.

To study the release in the artificial intestinal juice, the medium was completely replaced and the samples were extracted at one-hour intervals until the end of the test at 12 h. The volumes of the extracted samples were the same as in the previous stage, and supplementation with medium was performed in the same way. The samples obtained were filtered through a porous filter with a pore size of 0.45 µm, then diluted and read on an SPEKOL spectrophotometer at the specific wavelength of ketoprofen of 233 nm.

## 3. Results and Discussion

### 3.1. Analyses of the Solid K-β-CD Inclusion Complexes

#### 3.1.1. SEM Analysis

**Figure 2:**
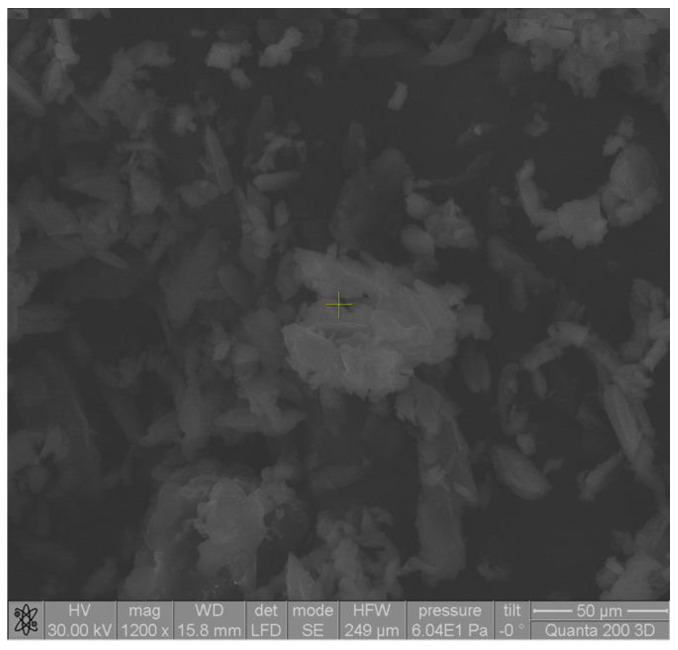
SEM image of ketoprofen–beta cyclodextrin (1:1) complex particles.

**Figure 3:**
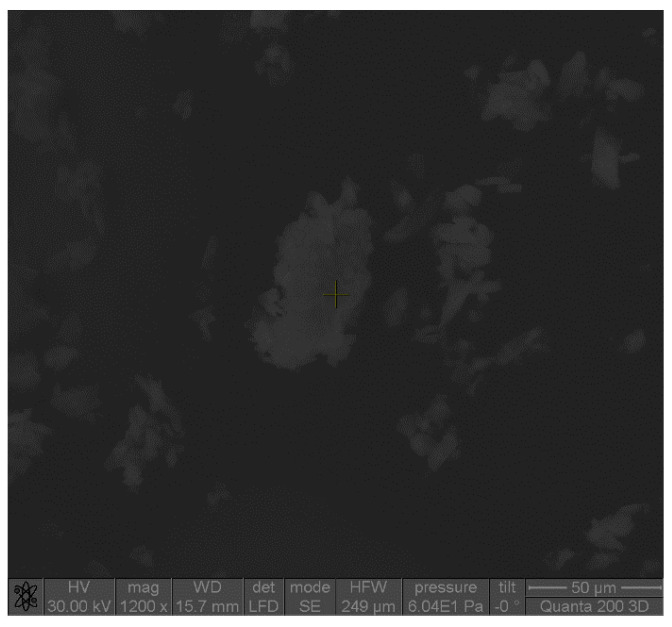
SEM image of ketoprofen–beta cyclodextrin (2:1) complex particles.

Microscopic analysis revealed that the shape of the complex particles was polyhedral with irregular sides (Figure 2). The particles were mostly associated in the form of aggregates in a thin layer due to the classical drying method (Figure 3). Microscopic images showed that the particles of the ketoprofen–beta cyclodextrin complexes were better individualized and presented uniform distributions in the samples as a result of the inclusion of ketoprofen molecules in the cyclodextrin cavities.

**Figure 4:**
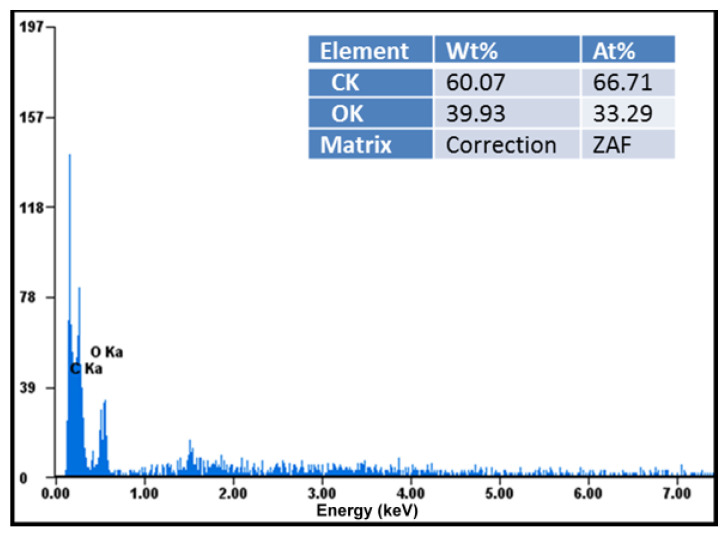
Elemental analysis of ketoprofen–beta cyclodextrin (1:1) complex particles.

**Figure 5:**
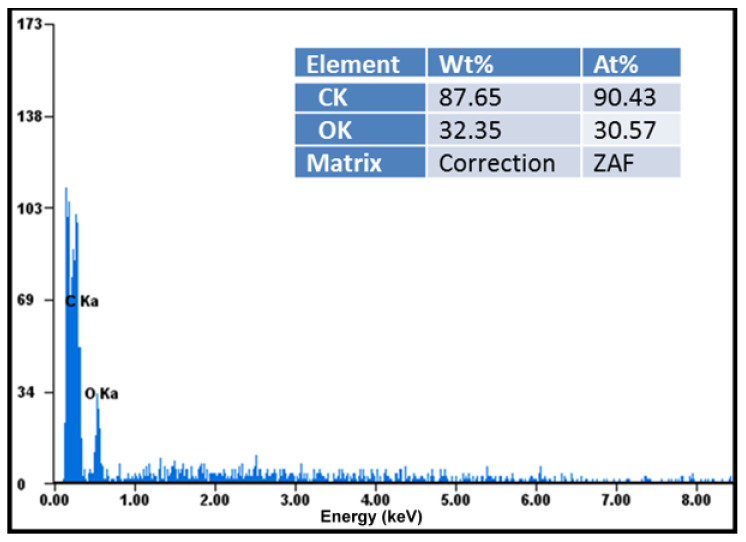
Elemental analysis of ketoprofen–beta cyclodextrin (2:1) complex particles.

Elemental analysis demonstrated that these compounds were chemically pure, with no chemical elements found other than carbon, oxygen in large quantities visible in the diagrams, and hydrogen in small quantities not indicated in the diagrams (Figure 4). EDAX analysis showed that the amount of carbon detected in the samples almost doubled in the case of the ketoprofen–beta cyclodextrin complexes (2:1) as a result of carbon added by the two molecules of ketoprofen included in the cyclodextrin structure (Figure 5).

#### 3.1.2. FTIR Analysis

The FT-IR spectra of ketoprofen, beta cyclodextrin, and the complexes ketoprofen–beta cyclodextrin (1:1) and ketoprofen–beta cyclodextrin (2:1) are presented in Figure 3. From the analysis of the FTIR spectra, it was observed that in the ranges 716–1655 cm^−1^ and 2939–3150 cm^−1^ differences appeared that were due to the characteristic groups involved in the bonds C=C, C=O, and –CH. Beta cyclodextrin showed key peaks at around 3412 cm^−1^ (O-H stretching), 2928 cm^−1^ (C-H stretching), and 1647 cm^−1^ (C-O-C bending). The C=C bonds registered an important peak for ketoprofen at 1599 cm^−1^.

**Figure 6:**
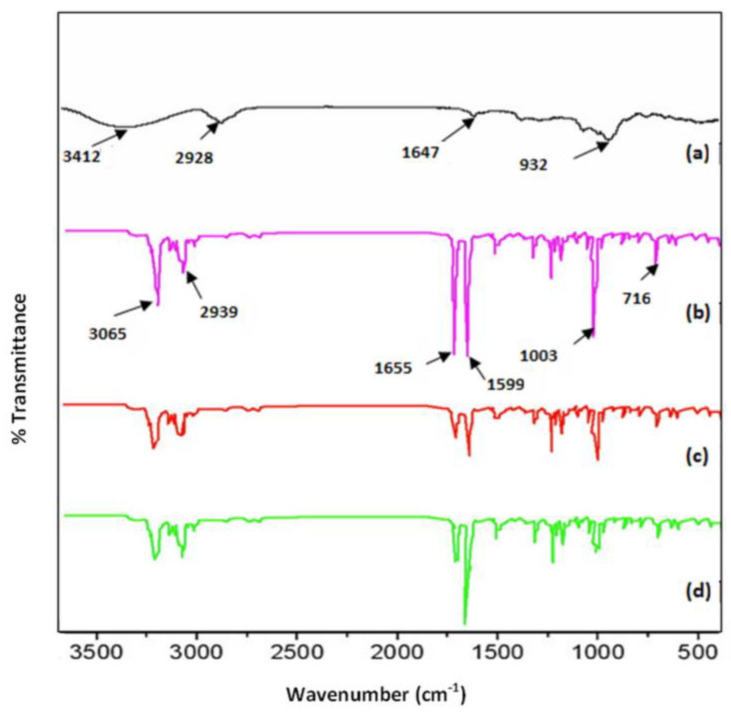
FTIR spectra: (**a**) β–cyclodextrin, (**b**) ketoprofen, (**c**) ketoprofen–β–cyclodextrin (1:1), and (**d**) ketoprofen–β–cyclodextrin (2:1).

The peak at 1599 cm^−1^, which was specific for the C=C bond, started at the ketoprofen–β-cyclodextrin (1:1) complex and ended at the ketoprofen–β-cyclodextrin (2:1) complex. The C=O bond decreased the peak at 1655 cm^−1^ for all complexes, and the -CH bond similarly decreased the peaks at 716 cm^−1^, 1003 cm^−1^, and 3065 cm^−1^, indicating the same ketoprofen inclusion efficiency (Figure 6).

#### 3.1.3. DSC Analysis

An analysis of the thermograms obtained for ketoprofen, beta cyclodextrin, ketoprofen–beta cyclodextrin (1:1), and ketoprofen–beta cyclodextrin (2:1) revealed the following information: the DSC curve of ketoprofen indicated the crystalline state of the substance with a characteristic melting point at 96 °C, and the DSC curve of β-cyclodextrin recorded a peak for the specific melting point at 298 °C.

Endothermic peaks characteristic of the loss of crystallization or adsorbed water were also observed on the curves of the complexes and β-cyclodextrin in the range of 40–110 °C. The residual moisture corresponded to the area < 100 °C. For the complexes, another interval of interest appeared, that of the water included in the cyclodextrin cavity, which corresponded to the area > 100 °C.

**Figure 7:**
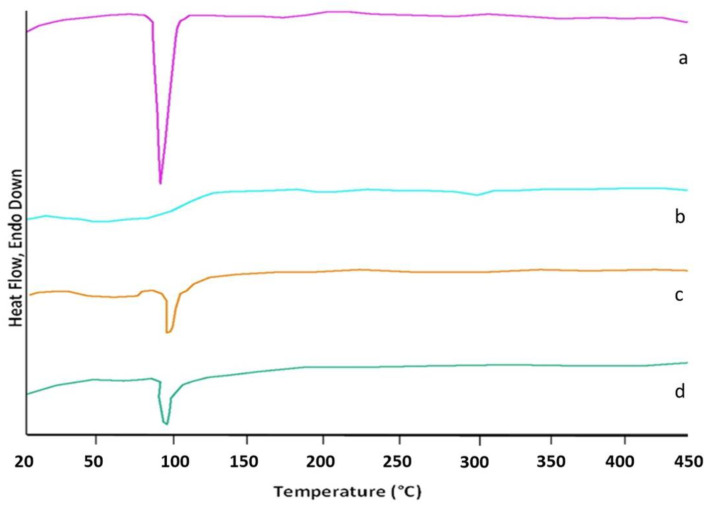
DSC thermograms of (**a**) K, (**b**) β-CD, (**c**) K-β-CD (1:1), and (**d**) K-β-CD (2:1).

In addition, in the DSC curve of β-cyclodextrin there was an endothermic peak that characterized the melting process of the substance at approximately 298 °C. In the two situations with the complexes, an essential decrease or total disappearance of the endothermic effects related to the melting of the formed ketoprofen–β-cyclodextrin complexes was observed, which indicated the interaction of ketoprofen with β-cyclodextrin and the formation of the complexes (Figure 7). The important peak areas were—862.42 for the ketoprofen, —35.21 for the β-cyclodextrin, —474.33 for the ketoprofen–β-cyclodextrin (1:1), and 543.32 for the ketoprofen–β-cyclodextrin (2:1). The Tpeak values for ketoprofen, β-cyclodextrin, ketoprofen–β-cyclodextrin (1:1), and ketoprofen–β-cyclodextrin (2:1) were 95.97 °C, 297.94 °C, 98.42 °C, and 97.81 °C. The enthalpy values for ketoprofen, β-cyclodextrin, ketoprofen–β-cyclodextrin (1:1), and ketoprofen–β-cyclodextrin (2:1) were 44.34 j/g, 1.81 j/g, 24.38 j/g, and 27.93 j/g.

#### 3.1.4. In Vitro Drug Dissolution Studies Using Complexes

**Figure 8:**
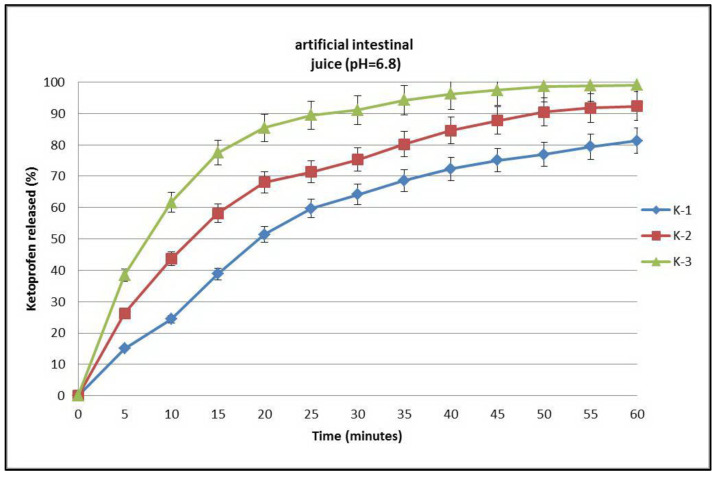
Dissolution profiles of ketoprofen (K-1), ketoprofen–β-cyclodextrin (2:1) (K-2), and ketoprofen–β-cyclodextrin (1:1) (K-3).

A graph of the dissolution curves of ketoprofen and its complexes with β-cyclodextrin in artificial intestinal juice (pH = 6.8) is presented in Figure 8, where it can be seen that after 60 min ketoprofen (curve K-1) registered a low curve due to its low solubility in the medium. In the case of the ketoprofen–β-cyclodextrin complexes (curves K-2 and K-3), dissolution was slightly improved, which can be observed in the dissolution curves (Figure 8) of both ketoprofen–β-cyclodextrin (2:1) (curve K-2) and ketoprofen–β-cyclodextrin (1:1) (curve K-3).

### 3.2. In Vitro Dissolution Studies Using Tablets

**Figure 9:**
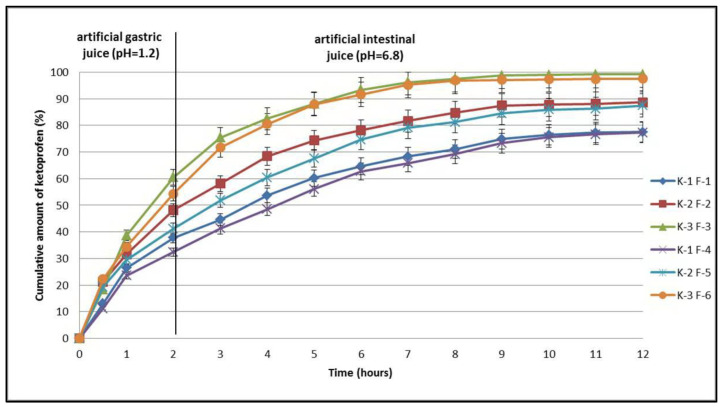
In vitro dissolution profiles of ketoprofen (K-1 F-1 and K-1 F-4), ketoprofen–β-CD (2:1) (K-2 F-2 and K-2 F-5), and ketoprofen–β-CD (1:1) (K-3 F-3 and K-3 F-6) from tablets tested.

Dissolution studies using compressed tablets revealed a prolonged release profile for the three substances, ketoprofen (K-1), the ketoprofen–beta cyclodextrin (2:1) complex (K-2), and the ketoprofen–beta cyclodextrin (1:1) complex (K-3). This phenomenon was dependent on the concentration of the matrix-forming polymers. The release kinetics were studied using two sets of tablets totaling six formulas, with the first set containing formulas F-1, F-2, and F-3 and the second set containing formulas F-4, F-5, and F-6. In the formulas of the first set of tablets, the concentration of Carbopol^®^ 971P NF was 4%, and in the second set the concentration of Carbopol^®^ 971P NF increased to 8%. Tablet dissolution studies in artificial gastric juice (pH = 1.2) recorded the highest concentration of the active substance released in the case of ketoprofen–beta cyclodextrin complex (1:1) sample K-3 F-3 (60.5%). The lowest concentration was recorded in the case of non-included ketoprofen sample K-1 F-1 (37.8%). When the amount of polymer increased in the tablet formula, the amount of the active substance decreased slightly, as shown by the results recorded in the cases of samples K-1 F-4 (32.4%) and K-3 F-6 (54.3%). For the formulas containing complexes of ketoprofen with beta cyclodextrin (2:1), the concentrations of the released active substance registered intermediate values for K-2 F-2 (48.2%) and K-2 F-5 (41.2%), as shown in Figure 9.

The second part of the dissolution study was carried out in artificial intestinal fluid (pH = 6.8), and in this case the release curves of the active substances recorded profiles specific to prolonged release (Figure 9). Five hours after the first set of tablets was introduced, the maximum amount of active substance released was recorded in the case of formula K-3 F-3 (88.1%) and the lowest was recorded in the case of formula K-1 F-1 (60.2%). Intermediate values were recorded for formula K-2 F-2 (74.3%). With an increase in the amount of polymer in the tablets, the values of the active substances released decreased as follows: K-1 F-1 (56.1%), K-2 F-5 (67.6%), and K-3 F-6 (87.9%). Seven hours after the first set of tablets was introduced, the maximum amount of active substance released was again recorded in the case of formula K-3 F-3 (96.2%) and the lowest was recorded in the case of formula K-1 F-1 (68.3%). Intermediate values were recorded for formula K-2 F-2 (81.6%). Increasing the amount of polymer in the tablets decreased the values of the active substances released as follows: K-1 F-1 (65.8%), K-2 F-5 (79.1%), and K-3 F-6 (95.2%).

According to the *European Pharmacopoeia, 11th edition*, a guarantee of almost total release of an active substance is given by a concentration of 80% [11]. In the studies conducted after 9 h, the amounts released from all formulas containing ketoprofen–beta cyclodextrin complexes were close to the maximum values. Thus, from the first set of tablets, the highest amount of active substance released was also recorded in the case of formula K-3 F-3 (98.7%) and the lowest was recorded in the case of formula K-1 F-1 (74.9%). Intermediate values continued to appear for formula K-2 F-2 (87.5%). The increase in the amount of polymer in the tablets continued to decrease the values of the active substances released as follows: K-1 F-1 (67.33%), K-2 F-5 (84.5%), and K-3 F-6 (97.1%). For formula K-3 F-6, the maximum value was reached after 9 h, and the values were maintained near the maximum until 12 h. Thus, from the first set of tablets, the maximum amount of active substance released was recorded after 11 h in the case of formula K-3 F-3 (99.1%) and the lowest amount was recorded in the case of formula K-1 F-1 (77.3%). Intermediate values were recorded for formula K-2 F-2 (88.6%). Increasing the amount of polymer in the second set of tablets decreased the values of the active substances released very slightly until 12 h as follows: K-1 F-1 (76.6%), K-2 F-5 (86.3%), and K-3 F-6 (97.5%) (Figure 9).

The obtained results confirm the effectiveness of including ketoprofen in complexes with beta cyclodextrin, which improved the release of the active substance from the tablets. The association of the active substance with increased amounts of a hydrophilic polymer such as Carbopol^®^ 971P NF confirmed its effectiveness in prolonging the release of the drug from the compressed tablets. These observations are consistent with data reviewed in the literature. Studies have shown that by combining the polymers Kollidon, Hypromellose, and Croscarmellose, the release of an active substance from tablets is controlled by the constant erosion of the tablets. As in other studies, the release of ketoprofen from the tablets was 98% (Figure 9) and depended on the type of complex it formed with cyclodextrin as well as the concentration of the polymers used [12,13,14,23,24,25,26,27,28,29,30]. Obtaining prolonged-release ketoprofen tablets ensures a reduction in the administered doses and increases patient compliance with treatment.

## 4. Theoretical Model

The polymer–drug system, a complex system utilized for controlled delivery of drugs, may be mathematically compared to a multifractal due to many characteristics of its behavior and structure. Presented below are some details that substantiate this analogy:

(i) Nonlinear dynamics and self-similarity over several scales [31,32]: The drug release mechanism in a polymer–drug system is influenced by several elements, including the interaction between the polymer and drug molecules, the architecture of the polymer–drug network, and alterations at both microscopic and macroscopic sizes. This behavior is nonlinear but demonstrates self-similarity across many observational scales; for instance, analogous patterns may emerge when investigations are conducted at differing degrees of granularity. A multifractal, in mathematics, is an entity that demonstrates self-similarity across many scales and possesses nonlinear characteristics, meaning distinct locations may display varying fractal dimensions. Consequently, the behavior of the polymer–drug system may be regarded as self-similar and nonlinear, exhibiting characteristics of multifractality.
(ii) Irregular drug distribution in polymers [32,33]: The polymer network structure is heterogeneous and can vary considerably, resulting in an irregular and non-uniform drug distribution. In a multifractal, the spatial distribution of points or components may be uneven and may display self-similarity across many levels of detail. The intricate architecture of polymers and the mechanisms of drug incorporation and release can be elucidated by mathematical principles related to multifractals.
(iii) Fluctuating fractal dimensions [34]: A significant aspect of multifractals is the fluctuation in fractal dimensions over various sizes. In the polymer–drug system, the physicochemical characteristics, including the drug’s diffusion rate, can fluctuate considerably based on the local circumstances of the polymer network. This behavioral diversity can be likened to the fractal dimensions of a multifractal, which exhibit varying dimensions based on the observational details of the system’s structures.
(iv) Intricate diffusion models [35]: Drug diffusion inside the polymer network may exhibit intricate patterns, where the movement of drug molecules is not only linear but is influenced by the network’s irregular structure. This phenomenon may be characterized using fractal mathematical models, which are employed to elucidate diffusion in irregular and complicated contexts. Fractality can characterize the variability in diffusion rates across several tiers of the polymer structure.
(v) Reliance on local circumstances and scalability impacts [36]: In a polymer–drug system, localized interactions between drug molecules and the polymer network can significantly impact the overall release process, with these effects being applicable across many structural layers. Scalability and reliance on local circumstances are defining features of multifractals, which exhibit self-similarity and sensitivity to small-scale factors. Thus, a correlation can be discerned between these characteristics of the system and multifractals.

Consequently, the intricate behavior and varied architecture of a polymer–drug combination may be effectively represented by mathematical notions pertaining to multifractals, enhancing our comprehension of the mechanisms involved in controlled drug administration.

In the following section, we will develop a theoretical model, considering that, both structurally and functionally, the polymer–drug system as a complex system can be assimilated to a multifractal mathematical object. Then, according to the Multifractal Theory of Motion [37], the polymer–drug dynamics can be described by continuous and non-differentiable curves (multifractal curves) so that the differential release equation can be written as follows (for details on obtaining this differential equation, please see [38]):

(1) $$\frac{\partial \rho }{\partial t}+V\frac{\partial \rho }{\partial z}-D\frac{\partial^{2}\rho }{\partial z^{2}}=0$$

where *t* is the non-multifractal time coordinate, *z* is the multifractal spatial coordinate, *V* is a constant that defines the rate associated with a multifractal–non-multifractal scale transition, *D* is the diffusion-type coefficient associated with the same transition, and *ρ* is the density of states of the polymer–drug system.

We note that D depends on the physical processes occurring during drug release from polymers and on the scale resolution and its properties.

The above model can describe the nonlinear behavior of a polymer–drug system that simultaneously follows release and diffusion processes, for example, through the transition from turbulent to laminar flow at various scale resolutions. From this perspective, the turbulence–laminar transition serves as a multifractal–non-multifractal scale transition, where V represents the drug release rate associated with that transition.

By varying the variables, the following equation can be obtained:

(2) $$\rho \left(z,t\right)=n\left(z,t\right)\exp(\frac{Vz}{2D}-\frac{V^{2}t}{4D})$$

Differential Equation (1) becomes [34]

(3) $$\frac{\partial n}{\partial t}=D\frac{\partial^{2}n}{\partial z^{2}}$$

Based on the initial and boundary conditions,

(4a) $$\rho \left(z,0\right)=\rho_{0}=const$$

(4b) $$\rho \left(0,t\right)=0$$

The boundary conditions corresponding to *n* are obtained, i.e.,

(5a) $$n\left(z,0\right)=n_{0}\exp(\frac{Vz}{2D})$$

(5b) $$n\left(0,t\right)=0$$

This problem can be reduced to a problem without boundary conditions by extending the solution to include the region z < 0, where n assumes an initial distribution that simulates the boundary condition for z = 0 at any time when t > 0.

Under these conditions, one can choose either

(6) $$n\left(z,0\right)=n(-z,0)$$

Therefore, for any extended region, the initial condition will be

(7) $$n\left(z,0\right)=\left\{\begin{array}{c} n_{0}\exp\left(\frac{Vz}{2D}\right),\;z<0\\ -n_{0}\exp\left(-\frac{Vz}{2D}\right),\;z>0 \end{array}\right.$$

The problem is how to solve the diffusion equation for an initially specified dimensional distribution in an unbounded medium. This can be solved by developing the solution as a Fourier integral.

(8) $$n\left(z,t\right)=\int_{-\infty }^{+\infty }\phi \left(k,t\right)\exp\left(ikz\right)dk$$

(9) $$\phi \left(k,t\right)=\frac{1}{2\pi }\int_{-\infty }^{+\infty }n(z^{\prime },t)\exp\left(-ikz^{\prime }\right)dz\prime$$

After substituting Differential Equation (3), we obtain

(10) $$\frac{\partial \phi }{\partial t}+k^{2}D\phi =0$$

which generates the following solution:

(11) $$\phi \left(k,t\right)=\phi \left(k,0\right)\exp(-k^{2}Dt)$$

where $\phi \left(k,0\right)$ is obtained by substituting the initial distribution (*n*) into Equation (9). Therefore, Equation (8) can be expressed as an integral over the entire initial distribution as follows:

(12) $$n\left(z,t\right)=\frac{1}{2\pi }\int_{-\infty }^{+\infty }\int_{-\infty }^{+\infty }n(z^{\prime },0)\exp\left(-k^{2}Dt\right)\exp\left[ik(z-z^{\prime })\right]dz\prime dk$$

Once the integral above is resolved with respect to *k*, solving for *n* becomes straightforward, leading to

(13) $$n\left(z,t\right)=\frac{1}{{\left(4\pi Dt\right)}^{\frac{1}{2}}}\int_{-\infty }^{+\infty }n(z^{\prime },0)\exp\left[-\frac{{\left(z-z^{\prime }\right)}^{2}}{4Dt}\right]dz\prime$$

Substituting Equation (7) into Equation (13) and computing the integral completes the solution. The result for $\rho \left(z,t\right)$ is given by the following equation:

(14) $$\rho \left(z,t\right)=\frac{n_{0}}{2}\exp\left(-\frac{Vt}{D}\right)+\frac{n_{0}}{2}\exp\left(-\frac{Vt}{D}\right)\cdot \left[\frac{erf(z-Vt)}{{\left(4Dt\right)}^{\frac{1}{2}}}+\frac{erf(z+Vt)}{{\left(4Dt\right)}^{\frac{1}{2}}}\right]$$

where $\mathrm{erf}\left(x\right)$ is the Laplace function given by

(15) $$\mathrm{erf}\left(x\right)=\frac{1}{2\sqrt{\pi }}\int_{0}^{\infty }\exp\left(-x^{2}\right)dx$$

The values of this function can be tabulated.

The rate of drug release from the polymer at z=0 is given by

(16) $$\begin{array}{ccc} I\left(t\right)=-\vec{j}\left(0,t\right){\hat{e}}_{z} & = & D{\left(\frac{\partial n}{\partial t}\right)}_{z=0}\\ & = & \rho_{0}\left\{\frac{V}{2}\cdot \left[1+\mathrm{erf}{\left(\frac{V^{2}t}{4D}\right)}^{\frac{1}{2}}+{\left(\frac{D}{\rho t}\right)}^{\frac{1}{2}}\exp\left(-\frac{V^{2}t}{4D}\right)\right]\right\} \end{array}$$

**Figure 10:**
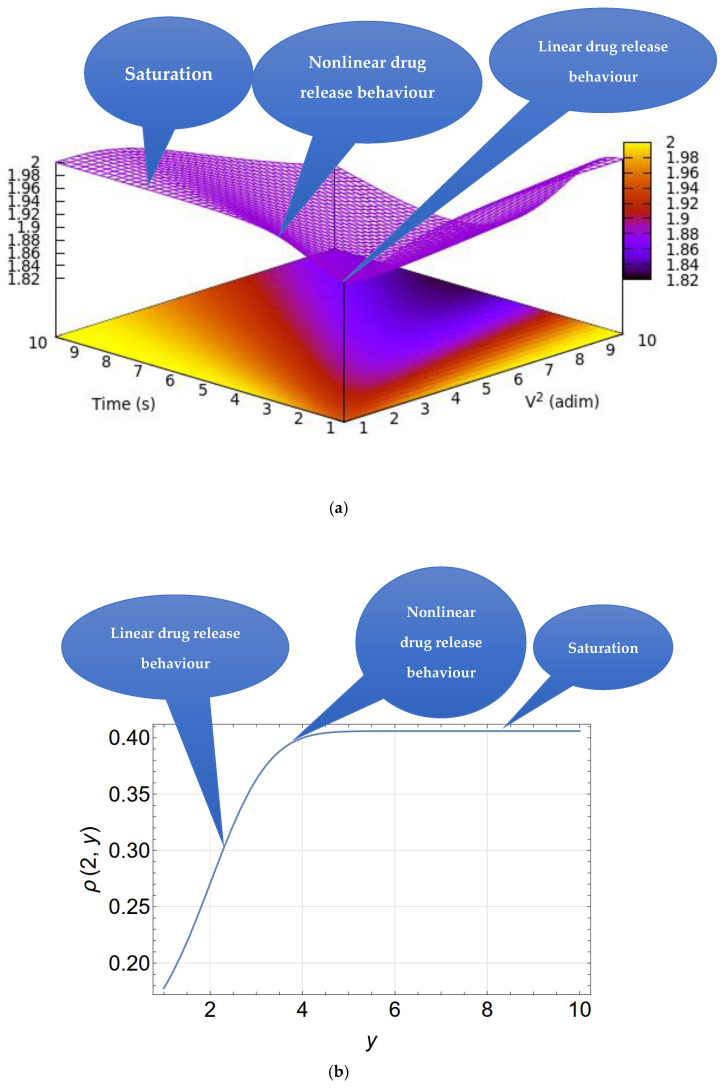
(**a**) A 3D representation of the drug release rate indicated by the color gradient as a function of time and *V*^2^ according to Equation (16), with *V*^2^ and time as reduced coordinates. (**b**) A drug release curve for a particular case (V = 2).

Figure 10a is a 3D depiction of the drug release rate versus time and V2. This provides a qualitative depiction of the drug release rate and shows that an increase in the quadratic rate may result in the highest release rate over a shorter period of time. However, over a longer period of time, the drug release rate may saturate (see also Figure 10b).

According to Equation (16), the diffusion current is infinite at t=0. This is because an infinite concentration gradient is artificially assumed at z=0 and t=0.

For $t\ll t_{e}=\frac{4D}{V^{2}}$, Equation (16) becomes

(17) $$I\left(t\right)=n_{0}\left[{\left(\frac{D}{\pi t}\right)}^{\frac{1}{2}}+\frac{V}{2}\right]$$

showing that the drug release rate is controlled by both the drug release rate and the diffusion velocity.

## 5. Conclusions

In this study, ketoprofen–β-cyclodextrin complexes were developed, demonstrating improved ketoprofen solubility. Using these complexes, polymer matrix tablets were successfully formulated, and they exhibited sustained release of ketoprofen over a 12 h period. In vitro studies revealed that the release of ketoprofen, both in its free form and from its β-cyclodextrin complexes, was influenced by the concentration of Carbopol^®^ in the formulations, as well as by the presence of other polymers such as Kollidon^®^, Hypromellose, and Croscarmellose, which played a key role in controlling the release kinetics through gradual tablet erosion.

The results showed that the K-3 F-3 tablets, containing ketoprofen–β-cyclodextrin complex (1:1), achieved more than 80% release within 4–7 h. In comparison, the K-2 F-2 tablets, formulated with the 2:1 complex, reached similar concentrations between 7 and 11 h, indicating a slower release rate. Furthermore, the K-3 F-6 tablets, formulated with the 1:1 ketoprofen–β-cyclodextrin complex and increased concentrations of retarding polymers, achieved 98% release after 12 h of testing. These results suggest an effective strategy for improving rheumatoid arthritis treatment by reducing the frequency of administration and enhancing patient acceptability. The obtained results are consistent with those previously reported in the literature [7,8,12,13,14,15].

A multifractal approach was employed to characterize the kinetics of drug release from the matrix. Within this framework, the polymer–drug system was considered a complex system, and the release dynamics were described by continuous and non-differentiable curves (multifractal curves). A comprehensive analysis of fractal size and scale resolution facilitated the characterization of the release dynamics of various ketoprofen variants.

## Data Availability

The original contributions of this study are included in this article; further inquiries can be directed to the corresponding author.

## Abbreviations

K	ketoprofen
K-β-CD	ketoprofen complexed with beta cyclodextrin
β-CD	beta cyclodextrin
NSAIDs	Nonsteroidal anti-inflammatory drugs
SEM	Scanning electron microscopy
FTIR	Fourier transform infrared spectroscopy
DSC	Differential Scanning Calorimetry
EDAX	Energy-Dispersive X-Ray Analysis
K-1 F-1	ketoprofen (tablet formula 1)
K-1 F-4	ketoprofen (tablet formula 4)
K-2 F-2	ketoprofen–beta cyclodextrin (2:1) (tablet formula 2)
K-2 F-5	ketoprofen–beta cyclodextrin (2:1) (tablet formula 5)
K-3 F-3	ketoprofen–beta cyclodextrin (1:1) (tablet formula 3)
K-3 F-6	ketoprofen–beta cyclodextrin (1:1) (tablet formula 6)
